# Clinical Relevance of Tubular Breast Carcinoma: Large Retrospective Study and Meta-Analysis

**DOI:** 10.3389/fonc.2021.653388

**Published:** 2021-04-29

**Authors:** Jasna Metovic, Alberto Bragoni, Simona Osella-Abate, Fulvio Borella, Chiara Benedetto, Maria Rosaria Gualano, Elena Olivero, Giacomo Scaioli, Roberta Siliquini, Pietro Maria Ferrando, Luca Bertero, Anna Sapino, Paola Cassoni, Isabella Castellano

**Affiliations:** ^1^Department of Oncology, University of Turin, Turin, Italy; ^2^Pathology Unit, Department of Medical Sciences, University of Turin, Turin, Italy; ^3^Department of Surgical Sciences, Gynecology and Obstetrics 1, University of Turin, Turin, Italy; ^4^Department of Public Health and Paediatric Sciences, University of Turin, Turin, Italy; ^5^Plastic Surgery Unit, Department of General and Specialistic Surgery, Città della Salute e della Scienza Hospital, Turin, Italy; ^6^Pathology Division, Candiolo Cancer Institute, FPO-IRCCS, Candiolo, Italy

**Keywords:** tubular carcinoma, meta-analysis, prognosis, hormone treatment, radiotherapy

## Abstract

**Background:** Tubular carcinoma (TC) is a low proliferative grade 1 (G1) breast cancer (BC). Despite its favorable outcome and allegedly lower aggressiveness, patients are treated like other luminal G1 BC, with radiotherapy (RT) and hormonal therapy (HT). We performed: (1) a retrospective study comparing a TC cohort and a control series of luminal G1 BC and (2) a systematic review and meta-analysis focused on TC outcome.

**Materials and Methods:** We selected a series of 572 G1 luminal BC patients [111 TC, 350 not otherwise specified (NOS), and 111 special-type (ST) BC] with follow-up and clinico-pathological data, who underwent local excision followed by RT at Città della Salute e della Scienza Hospital, Turin. Moreover, 22 and 13 studies were included in qualitative and quantitative meta-analysis, respectively.

**Results:** TCs were generally smaller (≤10 mm) (*P* < 0.001), with lower lymph node involvement (*P* < 0.001). TCs showed no local and/or distant recurrences, while 16 NOS and 2 ST relapsed (*P* = 0.036). Kaplan–Meier curves confirmed more favorable TC outcome (DFI: log-rank test *P* = 0.03). Meta-analysis data, including the results of our study, showed that the pooled DFI rate was 96.4 and 91.8% at 5 and 10 years, respectively. Meta-regression analyses did not show a significant influence of RT nor HT on the DFI at 10 years.

**Conclusions:** Compared to the other G1 BCs, TCs have an excellent outcome. The meta-analysis shows that TC recurrences are infrequent, and HT and RT have limited influence on prognosis. Hence, accurate diagnosis of TC subtype is critical to ensuring a tailored treatment approach.

## Introduction

The latest 2019 WHO edition categorizes breast cancers (BCs) in numerous entities (1) based on their specific histopathological characteristics.

Although these categories are associated with distinct clinical and prognostic implications, patients' management is mostly based on the evaluation of a well-defined set of markers, such as estrogen receptor (ER), progesterone receptor (PR), HER2, and Ki67, and the real meaning of the morphological model is often overlooked.

Tubular carcinoma (TC) is classified as “special-type” cancer and accounts for ~1–4% of all invasive BCs (2–4). TC represents a suitable example of an invasive tumor with an excellent outcome, but it is still treated like other BC with the same immune-phenotypic profile.

TC is a low proliferative grade 1 (G1) BC [according to Elston-Ellis classification (5)] belonging to the luminal A category, showing high levels of ER and PGR and lack of HER2.

Actually, several retrospective studies (6–9) reported that, among luminal G1 BCs, patients affected by TC have a significantly better prognosis, with a long-term outcome similar to that of age-matched women without BC (1).

In line with these findings, the guidelines of the Italian Association of Medical Oncology (2019) suggest avoiding systemic treatment after the diagnosis of a <1 cm TC. However, despite this recommendation, the clinical management of these lesions is often debated within the multidisciplinary tumor boards. Generally, in patients treated by conservative surgery, the management still remains radiotherapy (RT) plus 5 years of hormonal therapy (HT), like other luminal G1 BCs. Thus, a perception of an overtreatment is commonly acknowledged.

To address these issues, two types of analyses were performed: (1) a retrospective study comparing a cohort of patients affected by TC and a control series of luminal G1 BC, in order to assess possible clinico-pathological and prognostic differences and (2) a systematic review and meta-analysis focused on TC and its outcome.

## Materials and Methods

### Retrospective Study

#### Case Series

We selected 586 G1 luminal BC cases, from a retrospective series of BC patients who underwent conservative surgery at the Città della Salute e della Scienza Hospital, Turin, from January 1998 to December 2010. All patients received wide local excision followed by RT, while adjuvant systemic treatment was administrated based on patients' characteristics. The study was approved by the Research Ethics Committee for Human Biospecimen Utilization (Department of Medical Sciences—ChBU) of the University of Turin (n°9/2019). Written consent was not required considering the retrospective nature of the study. The study was conducted in accordance with The Code of Ethics of the World Medical Association (Declaration of Helsinki). All cases were de-identified, and all clinical-pathological data were accessed anonymously.

Patients with multifocal disease who underwent mastectomy and those with luminal HER2-positive BC were excluded, due to different treatment protocols.

Data regarding age at diagnosis, tumor size, vascular invasion, lymph node involvement, ER, PR, Ki-67, and treatment information were obtained from clinical charts and pathological reports. Follow-up data, including presence of local or distant recurrence, contralateral disease, death of disease (DOD), and death for other causes (DOC) were collected.

A cutoff value was set at 1% for ER and PR positivity (10) and at 20% for Ki67 proliferation index (11).

**Figure 1:**
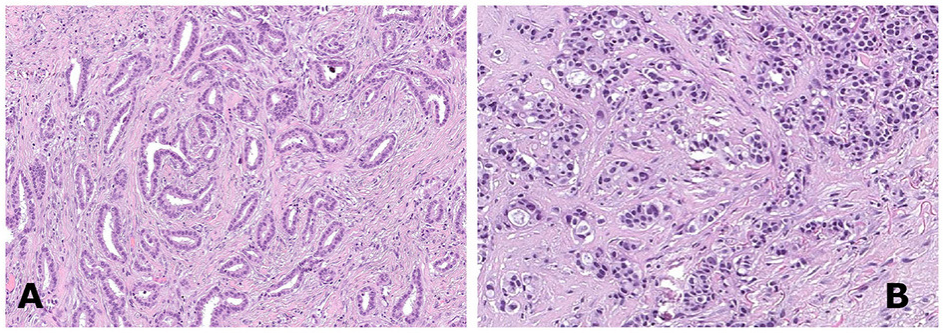
A case of tubular breast carcinoma composed of well-differentiated rounded to angulated tubular structures organized haphazardly [**(A)**, hematoxylin and eosin staining, 150×]. A case of invasive ductal carcinoma not otherwise specified (NOS), grade 1, demonstrating tumor cells arranged in clusters [**(B)**, hematoxylin and eosin staining, 150×].

Histological revision was performed according to WHO criteria by two of the authors (IC and JM). In particular, strict rules were applied to render the diagnosis of TC, based on (i) haphazard distribution of rounded and angulated tubules with open lumina in more than 90% of tumor tissue; (ii) a single layer of epithelial cells without significant atypia; and (iii) presence of abundant desmoplastic or fibro-elastotic stroma around the tubules (Figure 1).

Upon histological revision, we confirmed 572 cases as G1 luminal carcinomas, 111 pure TCs (19.5%), 350 (60%) non-special-type infiltrative carcinomas (NOS), and 111 (19.5%) special-type (ST) carcinomas, such as lobular, micropapillary, papillary, and cribriform BC.

#### Statistical Analysis

Patients' characteristics were compared using the Chi-square test for categorical variables and the *T-*test or ANOVA test for continuous variables, according to Bonferroni correction. The disease-free interval (DFI) was calculated from the date of surgical excision of the primary tumor to the date of the first relapse or last follow-up. Cases lost to follow-up were censored at the last visit time. Disease-specific survival (DSS) was calculated from the surgical excision date of the primary tumor to the date of breast cancer death. Survival distribution curves were plotted using the Kaplan–Meier method and compared with the log-rank test. Cox regression analyses were carried out on DFI to calculate crude and adjusted HRs and 95% CIs for the different groups. Local recurrence was defined as a tumor arising in the operated breast or in the axillary lymph nodes. The proportional hazard assumption was assessed with the Schoenfeld residuals, and this did not give reasons to suspect violation of this assumption. Cutoff values for the analyzed variables were set according to literature reports and/or the results of the log-likelihood ratio test. All statistical tests were two-sided, and *p* < 0.05 were considered significant. Statistical analyses were performed using Stata/SE13.0 Statistical Software (STATA, College Station, TX).

### Systematic Review and Meta-Analysis

#### Search Strategy and Literature Selection

A systematic literature review was performed using online electronic databases (PubMed and SCOPUS) for published papers until 1st March 2018. Search was performed using the following keywords: “tubular AND (breast OR mammary) AND (carcinoma OR carcinomas OR cancer OR cancers OR tumor OR tumors OR tumors OR tumor OR neoplasia OR neoplasm) AND (prognosis OR survival OR mortality OR relapse OR relapses).”

Considering only studies on breast TC, the following criteria were applied to select articles for further examination: (i) papers written in English language, (ii) studies conducted on humans, (iii) retrospective and prospective comparative studies and randomized controlled trials, and (iv) studies considering DFI as survival outcome.

Studies lacking the above-mentioned criteria were excluded from further investigation. When results could be mathematically combined, studies were also included in the meta-analysis.

The systematic review and meta-analysis were conducted and reported in accordance with the PRISMA (Preferred Reporting Items for Systematic Reviews and Meta-Analyses) statement (12).

#### Data Extraction

Data such as year of publication, study design, sample size, patients' characteristics including median age at diagnosis, median tumor diameter, presence of lymph node metastasis, type of surgery (mastectomy vs. conservative approach), type of adjuvant treatment (hormonal therapy, chemotherapy, radiotherapy), and 5/10-year DFI rates were independently extracted from the included studies by two investigators (AB and JM).

#### Statistical Analysis

Meta-analyses were performed to estimate the pooled 5/10-year DFI rates among women with TC. Meta-regression analyses were then carried out to perform a pooled analysis of factors affecting DFI rates, such as RT and HT. The Cochran Q and I^2^ were used to evaluate heterogeneity between the studies. To tackle potential sources of heterogeneity, the random-effect model was used to combine studies if heterogeneity was identified (Cochran Q *p* < 0.10 and I^2^ > 50%). The probability of publication bias was evaluated through the Egger's regression test and expressed by Funnel plots. Statistical analyses were performed using Comprehensive Meta-Analysis (CMA) software, version 3, Biostat Inc., Englewood, NJ, USA.

## Results

### Retrospective Study

The clinical-pathological characteristics of our case series are reported in Table 1.

**Table 1:** Clinical and pathological characteristics and outcome of the whole case series composed by G1 breast cancer patients, stratified according to histotype.

**Features**	**Tubular** **111 (A)**		**NOS** **350 (B)**		**Special type** **111 (C)**		***P*-value**	**Bonferroni** **correction**
	**N^°^**	**%**	**N^°^**	**%**	**N^°^**	**%**		
Age								
Median	56		60		64		0.007	/
Range	34–78		23–82		38–82			
Size								
≤ 10 mm	78	70.3	159	45.4	55	49.5	<0.001	A vs. B
>10 mm	33	29.7	191	54.6	56	50.5		*p <* 0.001, A vs. C *p =* 0.005
Vascular invasion								
No	108	97.2	312	89.1	105	94.5	0.011	A vs. B *p =* 0.008
Yes	3	2.7	38	10.9	6	5.4		
Lymph nodal involvement								
0	105	94.6	276	78.9	99	89.2	0.003	A vs. B *p <* 0.001
1–3	6	5.4	69	19.7	11	9.9		
4–9	0	/	5	1.4	0	/		
>9	0	/	0	/	1	0.9		
ER								
Mean % ± ds	91.8 ± 15.6		91.4 ± 14.1		93.2 ± 10.6		ns	
PR								
0	3	2.7	9	2.6	3	2.7	ns	
≥1	108	97.3	341	97.4	106	97.3		
Ki67 (missing 165)								
<20	72	97.3	251	94.4	59	88.1	ns	
≥20	2	2.7	15	5.6	8	11.9		
Hormonal treatment								
No	5	4.5	8	2.3	0	0	ns	/
Yes	106	95.5	342	97.7	111	100		
Chemotherapy								
No	111	100	326	93.1	103	92.8	0.017	A vs. B *p =* 0.018
Yes	0	/	24	6.9	8	7.2		

Tumor size was the main observed difference between TC and other G1 histotypes. Specifically, 70% of TC vs. 45 and 49.5% of G1-NOS and G1-ST, respectively, measured 10 mm or less (*P* < 0.001). Moreover, only 5% of TC cases showed lymph node involvement against 21% of G1-NOS and 11% of G1-ST (*P* < 0.001). In line with this, the presence of vascular invasion resulted to be different between TCs (3/111), G1-NOS (38/350), and G1-ST (6/111) (*P* = 0.011). Furthermore, the treatment approach varied between the groups: 7% of both G1-NOS and G1-ST patients received chemotherapy differently from TC cases which were treated with HT alone.

After a median follow-up of 9.3 years (7.2–11.1 years), we did not observe any recurrences (either local or distant) within the TCs, while we registered 16 events (eight local and eight distant recurrences) in G1 NOS BCs and 2 distant recurrences in G1 ST tumors (*P* = 0.036) (Table 2).

**Table 2:** Follow-up analysis of the whole case series stratified according to histotype.

	**Tubular** **111 (A)**		**NOS** **350 (B)**		**Special type** **111 (C)**		***P*-value**	**Bonferroni correction**
	**N°**	**%**	**N°**	**%**	**N°**	**%**		
Recurrence								
No	111	100	334	95.4	109	98.1	0.036	A vs. B, *p =* 0.021
Yes	0	/	16	4.6	2	1.8		
Type of recurrences								
Local	/	/	8	60	0	75	ns^*^	
Distant	/	/	8	40	2	25		
Metachronous contralateral								Not significant across all
No	108	97.3	348	99.4	107	96.4	0.046	
Yes	3	2.7	2	0.6	4	3.6		
Status								
Alive	104	93.7	329	94.0	103	92.8		
DOD	0	/	5	1.4	2	1.8	ns	
DOC	7	6.3	16	4.6	6	5.4		

* *ns, not significant; DOD, died of disease; DOC, died of other causes*.

In three patients with TC (measuring 15, 11, and 9 mm, respectively) contralateral BCs (two NOS and one lobular invasive carcinoma) were diagnosed. At follow-up, no patients with TC died of disease, while five patients with G1-NOS and two with G1-ST carcinomas were registered in the DOD category (Table 2).

**Figure 2:**
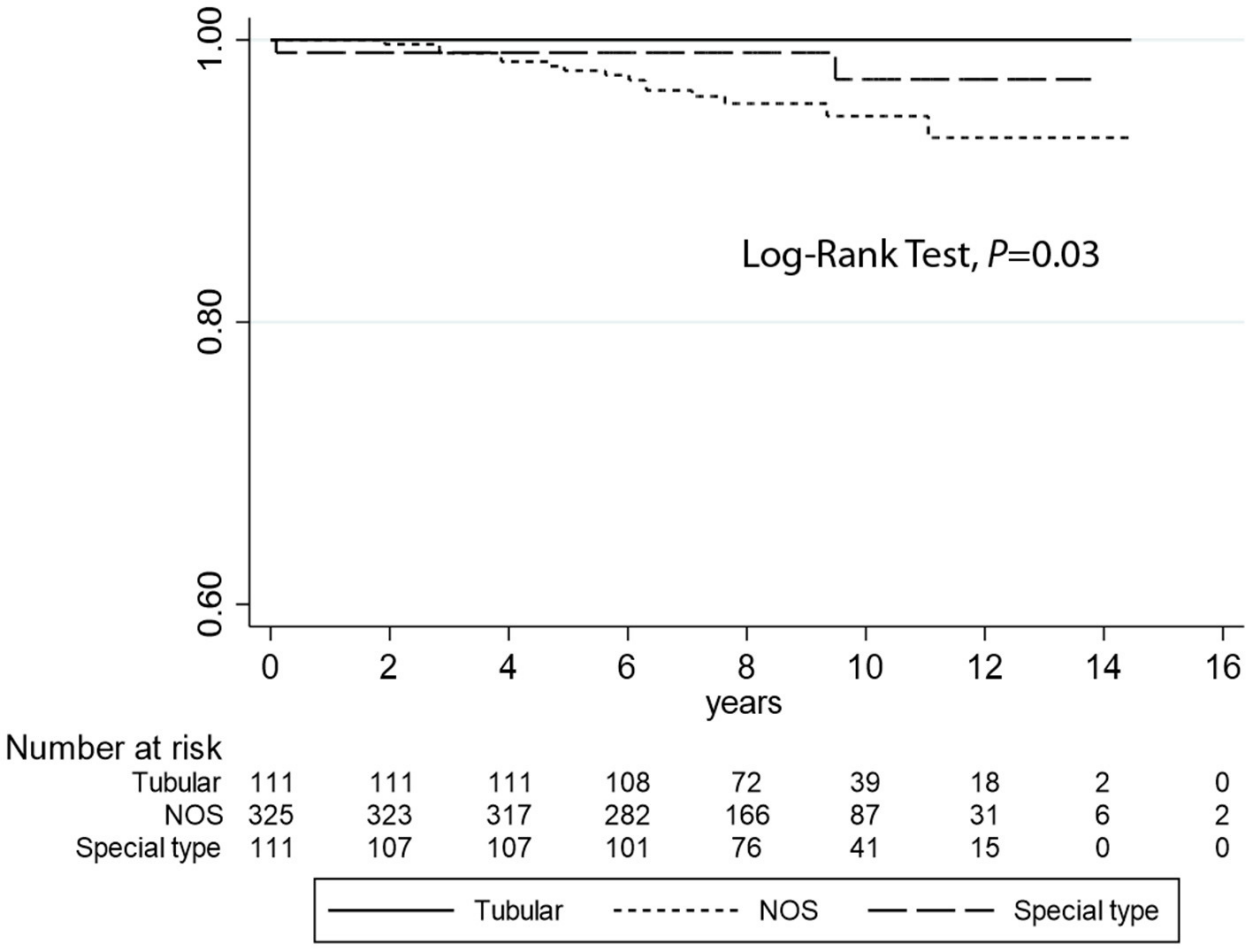
Kaplan–Meier estimates of DFI (log-rank test, *P* = 0.03) comparing tubular carcinomas with the other histotypes (not otherwise specified and special type breast cancers).

**Figure 3:**
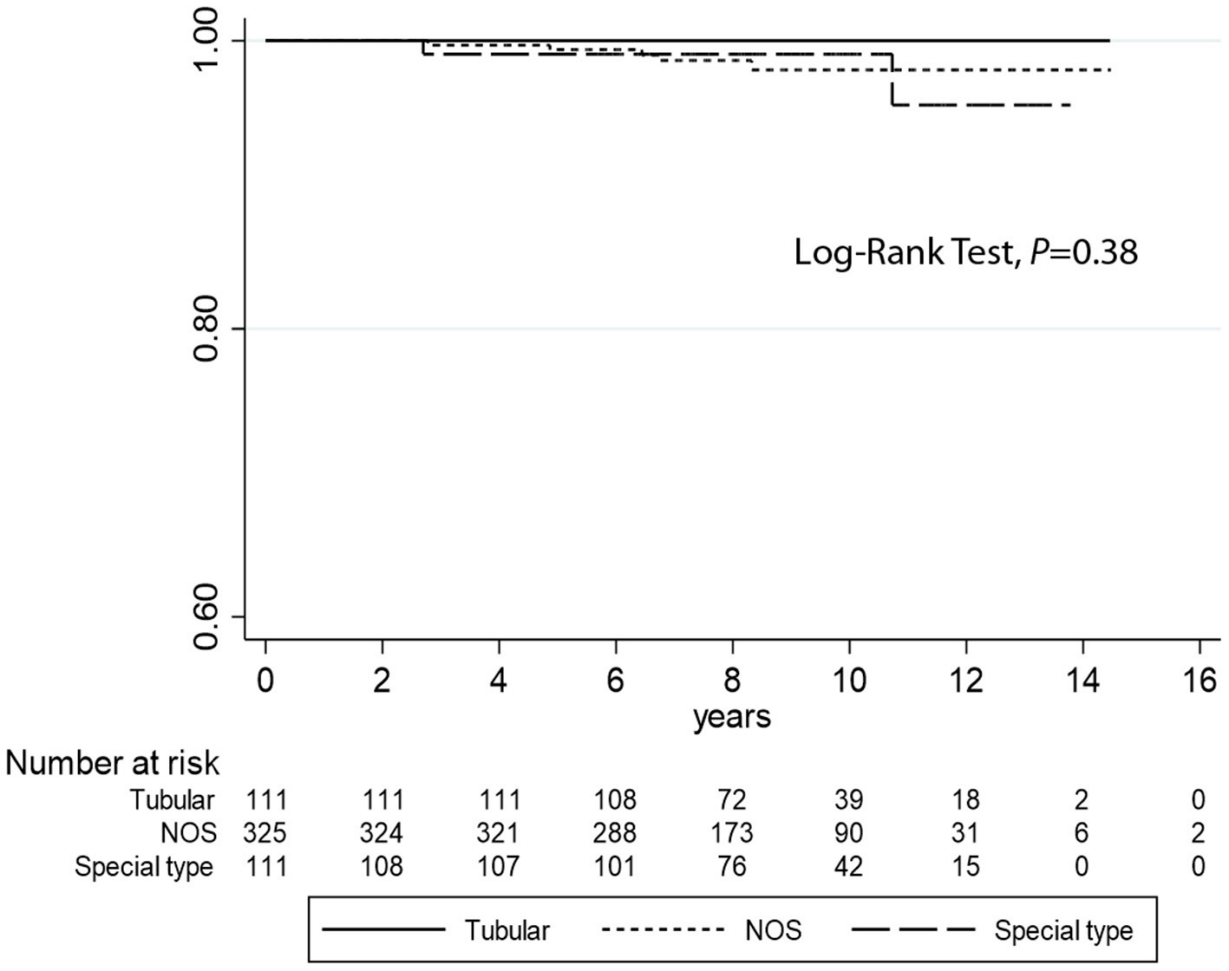
Kaplan–Meier estimates of DSS (log-rank test, *P* = 0.38) comparing tubular carcinomas with the other histotypes (not otherwise specified and special type breast cancers).

Kaplan–Meier curves (Figure 2) demonstrate that TC is associated with longer DFI (log-rank test *P* = 0.03) compared to the other histotypes. However, no statistical differences were observed regarding DSS analyses (log-rank test *P* = 0.38) (Figure 3). Univariate logistic regression analysis of DFI and DSS comparing TC and the other G1 histotypes did not reach statistical significance due to the low number of events (data not shown).

### Systematic Review

**Figure 4:**
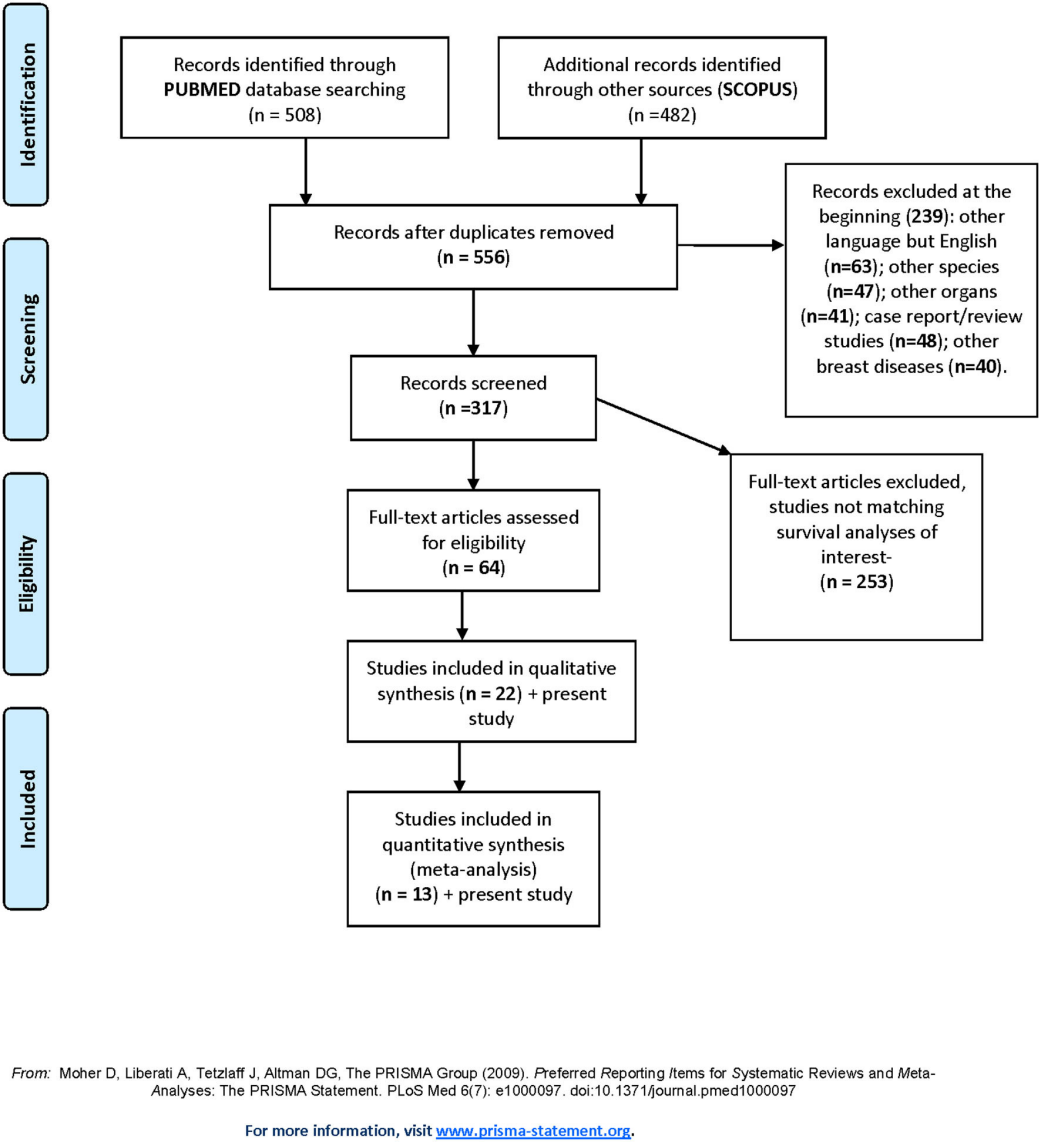
Prisma flowchart.

The results of the literature search and study selection process are summarized in PRISMA flow diagram (Figure 4).

A total of 556 titles and abstracts were obtained by electronic searches. Of these, 239 articles did not match the inclusion criteria described in the research strategy. Furthermore, 253 studies did not report the required survival analyses. Finally, 64 full-text articles were considered relevant and examined in detail. Following this comprehensive review, 22 studies were included in qualitative and 13 in quantitative synthesis for a total of 1,430 patients with TC (including our retrospective study). All the selected studies were retrospectively conducted. Most of the patients (1,378/1,430, 96.3%) were treated with conservative surgery, and ~70% of them also received adjuvant RT. The major features of the selected studies are summarized in Table 3. The present retrospective study was also included in the quantitative and qualitative analyses (Figure 4, Table 3).

**Table 3:** Characteristics of the studies considered in the meta-analysis.

**#**	**First author** **(year of publication)**	**Country**	**Study type**	**No. of** **cases**	**Median age** **(years)**	**Median FU** **(months)**	**BCS**	**HT**	**RT**	**DFI** **5 years**	**DFI** **10 years**
1	Winchester (1996) (13)	U.S.A.	Retrospective	50	54	58	21 (42%)	NS	16 (32%)	88%	NS
2	Haffty (1997) (14)	U.S.A.	Retrospective	21	49	126	NS	3 (14%)	NS	100%	100%
3	Diab (1999) (3)	U.S.A.	Retrospective	444	64	60	NS	408 (92%)	NS	94%	NS
4	Kader (2001) (15)	Canada	Retrospective	171	63	67	115 (67%)	29 (17%)	90 (53%)	95%	NS
5	Goldstein (2004) (16)	U.S.A.	Retrospective	32	54	125	32 (100%)	NS	32 (100%)	NS	96%
6	Leonard (2005) (17)	U.S.A.	Retrospective	44	67	65	44 (100%)	12 (27%)	9 (20%)	99%	91%
7	Vo (2007) (18)	U.S.A.	Retrospective	60	53	127	60 (100%)	5 (8%)	58 (97%)	97%	89%
8	Liu (2008) (6)	U.S.A.	Retrospective	36	56	84	66 (100%)	27 (41%)	66 (100%)	NS	99%
9	Colleoni (2011) (19)	Italy	Retrospective	83	NA	NA	73 (87%)	72 (86%)	74 (89%)	98.7%	NS
10	Bareggi (2012) (20)	Italy	Retrospective	75	58	NA	72 (96%)	33 (44%)	NS	97%	90%
11	Min (2013) (21)	Korea	Retrospective	70	47	52	65 (93%)	55 (78%)	65 (93%)	99%	92%
12	Cho (2018) (8)	Korea	Retrospective	205	48	70	174 (86%)	192 (94%)	167 (82%)	99%	NA
13	Thurman (2004) (22)	U.S.A.	Retrospective	28	51	120 (min.)	28 (100%)	0	50 and 61%^§^	82%	64%
**#**	Present study	Italy	Retrospective	111	56	112	111 (100%)	106 (95%)	111 (100%)	100%	100%
14	Lea (2014) (23)	Australia	Retrospective	223	57	96	125 (56%)	51 (22%)	100 (44%)	NS	NS
15	Fedko (2010) (24)	U.S.A.	Retrospective	105	60	62.4	62 (59%)	34 (33%)	51 (49%)	100%	NS
16	Oberman (1979) (25)	U.S.A.	Retrospective	25	44	90 (mean)	0	NS	1 (4%)	NS	NS
17	Bradford (1998) (26)	U.S.A.	Retrospective	63	57	48	38 (61%)	NS	21 (34%)	NS	NS
18	Günhan-Bilgen (2006) (27)	Turkey	Retrospective	32	51	67	20 (62%)	NS	NS	NS	NS
19	Rakha (2009) (7)	U.K.	Retrospective	102	54	127	79 (77%)	9 (9%)	34 (34%)	NS	NS
20	Boyan (2016) (28)	U.S.A.	Retrospective	57	60	72	41 (71%)	18 (31%) partial info	18 (31%) partial info	NS	NS
21	Carstens (1972) (29)	U.S.A.	Retrospective	35	51	60	1 (2%)	NS	4 (11%)	NS	NS
22	Stolnicu (2016) (30)	Romania	Retrospective	151	55	86	151 (100%)	NS	NS	NS	NS

*BCS, breast conserving surgery; HT, hormonal therapy; RT, radiotherapy; DFI, disease free interval; NS, not specified; Values are rounded up to or down to the nearest decimal*.

§ *50% axillary RT and 61% supraclavicular RT. Studies numbered 1 to 13 were inserted in quantitative synthesis, while all studies (1-22) were used for qualitative synthesis*.

### Meta-Analyses

The pooled DFI rate of TC was 96.1% (95% CI: 93.6–97.6%) at 5 years and 90.1% (95% CI: 82.1–94.8%) at 10 years. Egger's regression test showed no significant evidence for funnel plot asymmetry at 10 years (*P* = 0.11), while at 5 years it showed a potential publication bias (*P* = 0.03).

**Figure 5:**
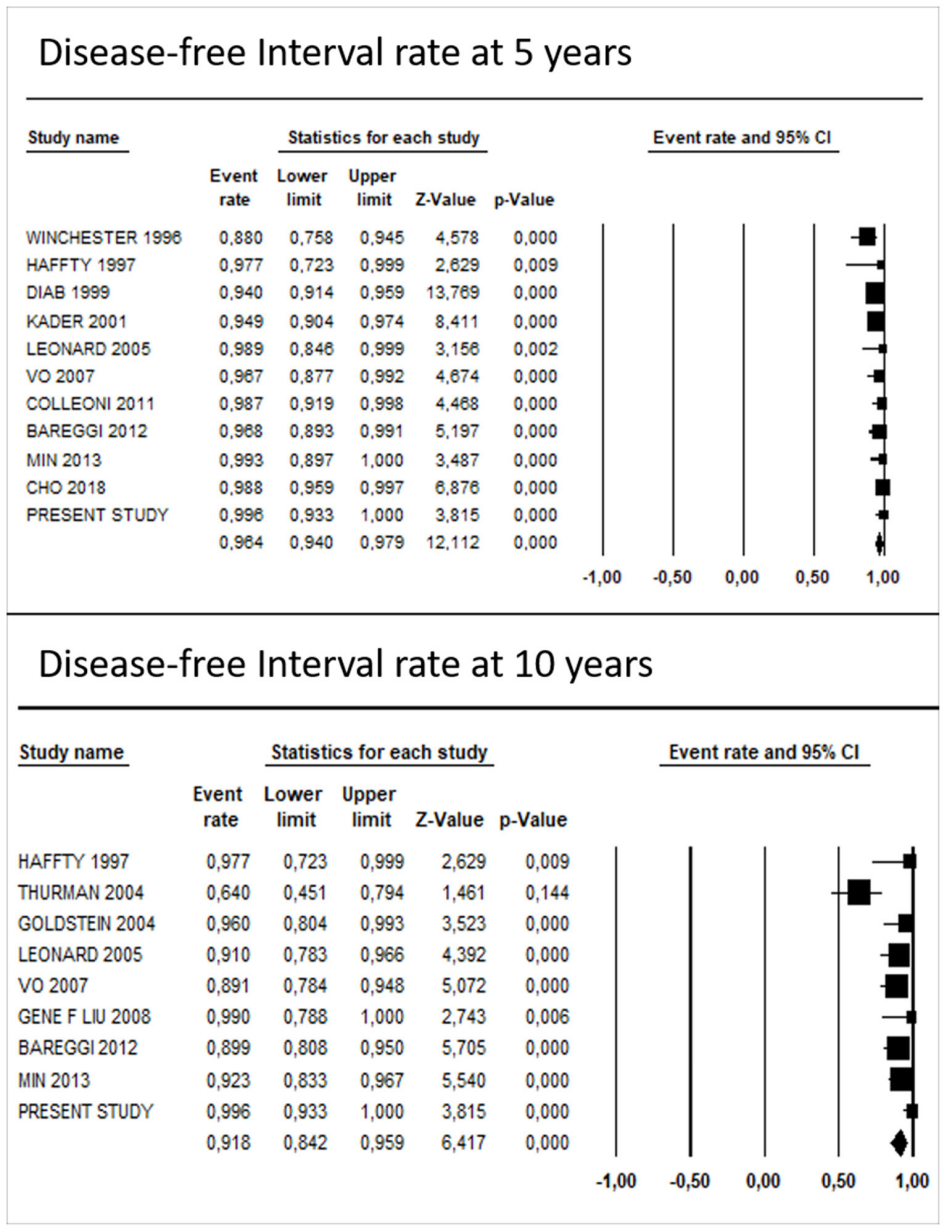
Pooled DFI rates at 5 and 10 years of studies assessing tubular carcinomas.

After including the results of our study in the analyses, the pooled survival rate was 96.4% (95% CI: 94.0–97.9%) and 91.8% (95% CI: 84.2–95.9%) at 5 and 10 years, respectively (Figure 5). The results of Egger's regression test showed a potential publication bias, both at 5 years (*P* = 0.01) and 10 years (*P* = 0.04).

#### Meta-Regression Analyses

Meta-regression analyses did not show a significant influence of RT on the DFI rates of TC at 10 years, after adjusting for median age and presence of lymph node metastasis, [1.78 (95% CI: −2.16; 5.72) and 2.00, (95% CI: −1.79; 5.79)] before and after including results of the present study, respectively. Also, HT did not show a significant association with the DFI rates [0.81 (95% CI: −0.63; 2.26) and 0.94 (95% CI: −0.48; 2.37)] (Table 4).

**Table 4:** Multivariable meta-regression analysis: potential influence of radiotherapy and hormonal therapy on disease-free survival rates of tubular carcinoma at 10 years.

	Multivariable analysis^*^			Multivariable analysis^*^ **(present study included)**		
**DFI rate**	**Coefficient**	***P***	**95% CI**	**Coefficient**	***P***	**95% CI**
Radiotherapy rate	1.78	0.38	−2.16; 5.72	2.00	0.30	−1.79; 5.79
Hormonal therapy	0.81	0.27	−0.63; 2.26	0.94	0.19	−0.48; 2.37

* *Adjusted for median age and lymph node metastasis rate*.

## Discussion

TC is special-type invasive BC, characterized by a well-differentiated histology and an excellent outcome. The diagnosis of TC is rendered only in case of tubule formation being demonstrated in 90% of the tumor. To date, generally, patients with TC undergo both HT and RT, like other luminal BCs. Despite a perception of an overtreatment, the proposed de-escalation of TC management is still to be translated to the daily practice. This is mainly due to different clinical experience and presence of heterogeneous literature data, including small case series, different clinical approaches, different follow-up lengths, and lack of strict histological review.

To tackle this open issue, we performed a retrospective analysis of our large institutional series of G1 BC focusing on TC outcome and we comprehensively analyzed the available data through a systematic review of literature and meta-analysis.

Our results confirmed the small size (generally <10 mm) of TC and their excellent outcome, in line with literature data (3, 31). Moreover, in our case series no local/distant recurrences or DOD were observed.

Actually, compared to invasive ductal carcinoma, TC is more likely to be detected on mammographic screening (7), mainly due to its typical dense fibro-elastotic stroma that is promptly visualized on mammography (32).

Considering that TC has limited impact on outcome (3, 8, 18, 33–36), several groups hypothesized that its detection could represent an example of overdiagnosis (37). However, Aulmann et al. (38) provided the molecular evidence for a direct clonal relationship of flat epithelial atypia and low-grade ductal carcinoma *in situ* with TC, indicating their precursor role. Furthermore, it is important to note that many NOS BC have a tubular component suggesting that TCs could de-differentiate into more aggressive cancers if left unresected. Moreover, almost 5% of TC patients show metastatic disease in axillary lymph nodes (24, 39) as also demonstrated in our study; thus, they seem to harbor an intrinsic malignant potential All these data confirm the importance of surgical treatment (37). On the other hand, even in the presence of lymph node involvement, TC showed no consequences on outcome, suggesting that omission of axillary surgery, even after metastatic sentinel lymph node, may be a valid choice (40, 41).

We would like to emphasize the importance of distinguishing TC from other similar BC histotypes, following strict morphological criteria. In fact, despite their histopathological similarity with G1 NOS BC, TCs differed in terms of size, lymph nodal status, and angioinvasion, thus supporting their true biological peculiarity.

Concerning adjuvant treatments, despite the more common use of chemotherapy, patients with both G1-NOS and ST BCs recurred in ~5 and 2% of cases, respectively, unlike TC which had a more favorable outcome in terms of DFI (log rank test *P* = 0.03).

These results are in agreement with the comprehensive systematic review and meta-analysis here performed which included 22 studies and data of 1,430 patients. The pooled 5- and 10-year DFI rates (96.4 and 92%, respectively) support the extremely favorable outcome of TC. Moreover, our multivariable meta-regression results suggest that neither HT (*P* = 0.27) nor RT (*P* = 0.38) has a significant association with DFI, after adjusting for median age and presence of lymph node metastases.

In line with our data (3, 7, 42), some studies demonstrated that the survival of patients with TCs after conserving surgery and RT is similar to the general population, suggesting that these patients may be safely spared the side effects and costs of HT (43, 44).

On the other hand, TC is associated with an increased number of contralateral disease (7, 8, 13, 27). In our series, we found 3/111 cases with metachronous contralateral tumor; all of them measured 10 mm or more.

These data suggest that HT, as proposed by official guidelines [AIOM (45) and NCCN (46)], may still have a role in TC with larger diameter, although it may be omitted in TC sized <1 cm.

Regarding RT, although a de-escalation has been proposed in low-risk BC patients (47), its employment in TC is a matter of debate. A recent work by Wu et al. (48) proposed omitting RT in patients aged ≥65 years, while in another study by Chen et al. (49) the authors suggested a potential benefit of RT in patients aged ≤ 50 years. In our series, since all the patients were treated with RT, it was not possible to evaluate its effect on prognosis. However, taking into consideration the complete absence of local relapses and that RT showed no significant impact on outcome by meta-regression analysis, its omission could be a possible choice, to be investigated in future studies.

In conclusion, our study confirms that patients with TC have an excellent outcome, superior to other G1 BCs. Moreover, to the best of our knowledge, this is the first meta-analysis aimed at specifically evaluating the characteristics and outcome of TC, showing that recurrences are infrequent and that both HT and RT have limited influence on prognosis. Thus, accurate histopathological diagnosis of the TC subtype is crucial to providing correct prognostic information, paving the way for treatment personalization.

## Data Availability Statement

The raw data supporting the conclusions of this article will be made available by the authors, without undue reservation.

## Author Contributions

IC contributed to conception and design of the study. Data acquisition was performed by JM, AB, FB, and PF. JM and AB organized the database. Data analyses were performed by SO-A, MG, EO, and GS. Interpretation of the data was executed by IC, JM, SO-A, LB, MG, EO, and RS. Project supervision was offered by IC, PC, and AS. The first draft of the manuscript was written by IC, JM, SO-A, MG, and EO. All authors contributed to manuscript revision and read and approved the submitted version.

## Conflict of Interest

The authors declare that the research was conducted in the absence of any commercial or financial relationships that could be construed as a potential conflict of interest.
